# PDLIM7 and CDH18 regulate the turnover of MDM2 during CDK4/6 inhibitor therapy-induced senescence

**DOI:** 10.1038/s41388-018-0332-y

**Published:** 2018-05-23

**Authors:** Mary E. Klein, Mark A. Dickson, Cristina Antonescu, Li-Xuan Qin, Scott J. Dooley, Afsar Barlas, Katia Manova, Gary K. Schwartz, Aimee M. Crago, Samuel Singer, Andrew Koff, William D. Tap

**Affiliations:** 1 The Louis V. Gerstner Graduate School of Biomedical Science, Memorial Sloan Kettering Cancer Center, 1275 York Avenue, New York, NY 10065 USA; 2 The Programs in MolecularMemorial Sloan Kettering Cancer Center, 1275 York Avenue, New York, NY 10065 USA; 3 Departments of Medicine, Memorial Sloan Kettering Cancer Center, 1275 York Avenue, New York, NY 10065 USA; 4 Departments of Medicine, Weill Cornell Medical College, New York, NY 10021 USA; 5 Departments of Pathology, Memorial Sloan Kettering Cancer Center, 1275 York Avenue, New York, NY 10065 USA; 6 Departments of Epidemiology and Biostatistics, Memorial Sloan Kettering Cancer Center, 1275 York Avenue, New York, NY 10065 USA; 7 Center for Advanced Retinal and Ocular Therapeutics, Department of Ophthalmology, Perelman School of Medicine, University of Pennsylvania, Philadelphia, PA 19104 USA; 8 Developmental Biology, Memorial Sloan Kettering Cancer Center, 1275 York Avenue, New York, NY 10065 USA; 9 Division of Hematology and Oncology, Columbia University Medical Center, Herbert Irving Pavilion 9th floor, 161 Fort Washington Avenue, New York, NY 10032 USA; 10 Departments of Surgery, Memorial Sloan Kettering Cancer Center, 1275 York Avenue, New York, NY 10065 USA; 11 Departments of Surgery, Weill Cornell Medical College, New York, NY 10021 USA

## Abstract

CDK4/6 inhibitors are being used to treat a variety of human malignancies. In well-differentiated and dedifferentiated liposarcoma their clinical promise is associated with their ability to downregulate the MDM2 protein. The downregulation of MDM2 following treatment with CDK4/6 inhibitors also induces many cultured tumor cell lines derived from different types of malignancies to progress from quiescence into senescence. Here we used cultured human cell lines and defined a role for PDLIM7 and CDH18, regulating MDM2 protein in CDK4/6 inhibitor-treated cells. Materials from our previous phase II trials with palbociclib were then used to demonstrate that expression of CDH18 protein was associated with response, measured as both progression-free survival and overall survival. This supports the hypothesis that the biologic transition from quiescence to senescence has clinical relevance for this class of drugs.

## Introduction

The commitment to cell proliferation is initiated when extracellular signals converge at the cell cycle and induce the expression of D-type cyclins, their association with CDK4 and/or CDK6, and the activation of the holoenzyme complex [1–3]. The cyclin D-associated kinases are necessary for the proliferation of Rb-positive cells because they initiate the phosphorylation-dependent cascade that inactivates this tumor suppressor [2, 4]. Unchecked proliferation of Rb-positive tumor cells is commonly associated with mutations that dysregulate this pathway: including the overexpression of D-type cyclins, the mutation or overexpression of CDK4, or mutations in the INK4 family of CDK inhibitors [3, 5, 6]. The importance of cyclin D holoenzymes for inactivation of Rb and the development of cancer in mice prompted the development of CDK4/6 inhibitors to treat a variety of neoplasms [7, 8]. These inhibitors have had success, both as a monotherapy and in combination [9].

Multiple cellular mechanisms have been advanced to account for the clinical activity of CDK4/6 inhibitors (reviewed in Klein et al., Cancer Cell in press). Many Rb-positive cells exit the cell cycle after CDK4/6 inhibition [10–16]. Resistance to these drugs, either acquired or innate, has been suggested to be due to a failure of the tumor cell to exit in response to the drug, linked to a failure to mobilize cells of the tumor microenvironment, or associated with the inability of the tumor cell to progress from reversible quiescence into more permanent senescence.

The decision of a tumor cell to senesce after CDK4/6 inhibition is made after the cell has withdrawn from the cell cycle. This previously unrecognized transition, now called senescence after growth arrest or SAGA, is triggered in the CDK4/6 inhibitor-induced quiescent cell by the loss of MDM2 protein and increased focal localization of the chromatin-remodeling enzyme ATRX [17, 18]. Palbociclib (also known as PD0332991)-induced senescence is not due to increased p53 [13, 18], nor is it associated with increased DNA damage [17].

The PD0332991-induced downregulation of MDM2 and entry into senescence is observed in a number of different types of cancer cell lines, including those derived from well-differentiated and dedifferentiated liposarcoma (WD/DDLS), breast cancer, non-small cell lung cancer, and glioma [18]. In a small pilot study of seven patients with WD/DDLS treated with palbociclib, the downregulation of MDM2, but not the absolute amount of the protein, also associated with how patients respond to the drug [18]. Thus, to understand how palbociclib improves patient outcomes it is important to understand how MDM2 is regulated in PD0332991-treated cells.

A number of cell type and signal-specific regulatory pathways can impact upon the accumulation of MDM2 protein (reviewed in ref. [19]). During SAGA, intrinsic E3 ligase activity is necessary for the downregulation of MDM2 [18]. HAUSP is a deubiquitinase that binds to MDM2 and removes ubiquitin from it, stabilizing the protein and allowing it to ubiquitinate other substrates [20, 21]. However, HAUSP dissociates from MDM2 as cells exit the cell cycle following palbociclib treatment, indicating that HAUSP does not play a role in whether quiescent cells downregulate MDM2 and proceed into senescence [18]. Thus, we set out to identify what stabilizes MDM2 protein in quiescent cells.

After attempting to knockdown five different genes whose proteins had been previously shown to inhibit MDM2 turnover [19], we show that one, PDLIM7, a PDZ and LIM domain-containing protein that binds to MDM2, is needed to stabilize MDM2 and prevent PD0332991-induced senescence. PDLIM7 was previously shown to inhibit MDM2 autoubiquitination and allow MDM2 to ubiquitinate p53 [22]. In cells that undergo senescence following PD0332991 treatment, we found that PDLIM7 was sequestered away from MDM2 by association with a type II cadherin, CDH18. In addition, both progression-free survival (PFS) and overall survival (OS) was significantly extended in patients with WD/DDLS tumors that are CDH18-positive and whom received palbociclib as a single agent in phase II clinical trials [23, 24]. This not only establishes that CDH18 and PDLIM7 impact on the regulation of MDM2 in PD0332991-induced quiescent cells, it reinforces the notion that SAGA contributes to the clinical outcome of patients treated with palbociclib.

## Results

### PDLIM7 knockdown allows PD0332991 to induce MDM2 turnover and senescence in cells that would otherwise quiesce

To gain insight into how MDM2 turnover was prevented in PD0332991-induced quiescent cells we carried out a small-targeted knockdown screen where we attempted to individually reduce expression of CREBBP, NEDD4-1, PDLIM7, PRKCD, or p300, all previously reported to regulate MDM2 auto-ubiqutination [19]. Parental WD/DDLS-derived LS8107 cells exit the cell cycle after PD0332991 treatment but MDM2 levels do not decrease and the cells do not undergo senescence. Throughout this manuscript we call such cells that when treated with PD0332991 do not undergo senescence as “non-responders”. In contrast, a ‘responder cell’ will exit the cell cycle after treatment with PD0332991 and progress into senescence. Knocking down MDM2 in PD0332991-induced quiescent non-responder cells will induce senescence [18].

**Fig. 1:**
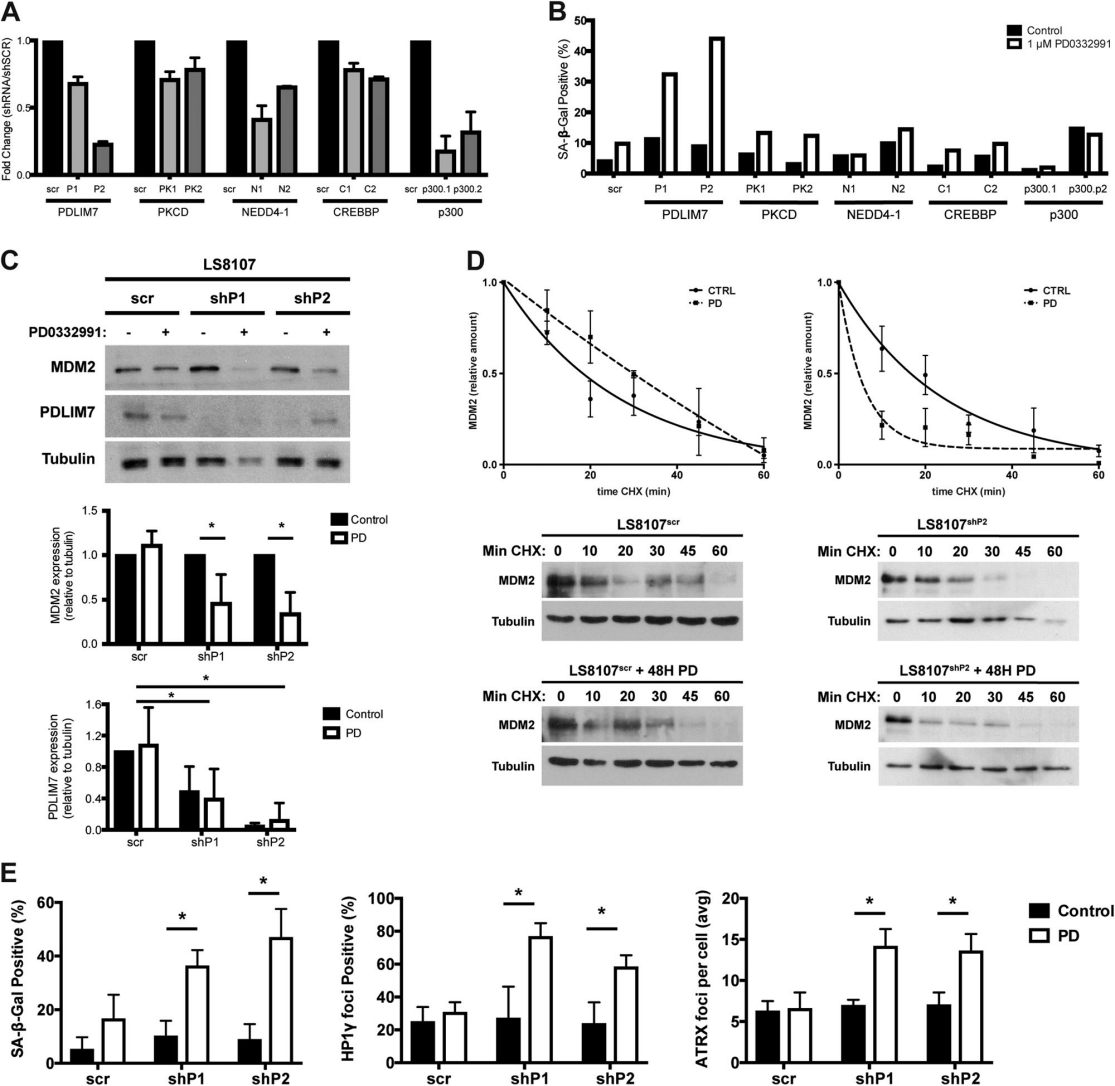
PDLIM7 knockdown allows PD0332991 to induce MDM2 turnover and senescence in LS8107 cells. **a** The non-responder cell line LS8107 was transduced with short hairpin expressing lentiviruses targeting either the indicated gene product or containing a non-specific sequence (scr), and selected in puromycin for 5 days. **a** Cells were harvested, RNA extracted, and qRT-PCR was performed to measure mRNA expression of the individual targets and quantitiated relative to the expression level in the LS8107 cells expressing the scr sequence. β-actin was used as a normalization control. The mean and standard deviation of three technical replicates is plotted. **b** Cells in **a** were treated with PD0332991 (PD) for 7 days and the number of cells staining for SA-β-gal quantified. **c** The cells transduced with the two different PDLIM7 knockdown lentiviral vectors (shP1 or shP2) or a non-specific vector (scr) were treated as in **b**, and MDM2 and PDLIM7 protein levels were detected using immunoblot. Tubulin served as a loading control. Top, a representative image is shown. Bottom, expression was quantified using densitometry and the mean and standard deviation of four independent experiments is shown. **d** LS8107^scr^ and LS8107^shP2^ cells were treated as described in **b** and then exposed to 75 μg/mL cyclohexamide (CHX) for the time (min) indicated. MDM2 and tubulin were measured by immunoblot. A representative image is shown below and the relative amounts were quantified from two independent experiments (mean ± standard error of measurement) above. **e** The number of cells staining for SA-β-gal, HP1γ foci, and the number of ATRX foci per cell were quantified and the mean and standard deviation of four independent experiments is plotted

Two independent lentiviral shRNA-targeting vectors efficiently reduced the level of PDLIM7, NEDD4-1, and p300 mRNAs (Fig. 1a). However, only in those in which PDLIM7 was reduced would PD0332991 treatment strongly induce the accumulation of senescence-associated beta-galactosidase (SA-β-gal), a marker of senescent cells (Fig. 1b). We confirmed that reducing PDLIM7 with the P2 shRNA also promoted the accumulation of SA-β-gal in another non-responder cell line, LS8313 (Supplemental Fig. 1). Furthermore, enforced expression of a his-biotin tagged mutant PDLIM7 in which the nucleic acid sequence was mutated to prevent interaction with the shRNA without affecting the protein-coding sequence prevented significant accumulation of SA-β-gal following treatment with PD0332991 (Supplemental Fig. 1).

To confirm that reducing PDLIM7 altered the outcome of PD0332991-induced cell cycle exit we expanded our analysis to other markers of senescence. Seven days after the addition of PD0332991 to cell lines stably expressing either of the two different shRNAs targeting PDLIM7, LS8107^shP1^, or LS8107^shP2^, MDM2 levels decreased compared with a cell line stably expressing a scrambled shRNA, LS8107^scr^ (Fig. 1c). Reduced levels of MDM2 were associated with the accelerated turnover of MDM2 in the LS8107^shP2^ cells treated with cyclohexamide (Fig. 1d). Similar results were observed in the LS8107^shP1^ cells. Furthermore, the accumulation of SA-β-gal-positive cells, senescence-associated HP1γ heterochromatic foci (SAHF)-positive cells, and the number of nuclear ATRX foci were increased after the addition of PD0332991 in both LS8107^shP1^ and LS8107^shP2^ cells but not LS8107^scr^ cells (Fig. 1e). Thus, reducing PDLIM7 expression can alter the outcome of PD0332991-induced cell cycle exit, converting non-responder cells into responder cells.

### The association of PDLIM7 with MDM2 is cell line and condition dependent

**Fig. 2:**
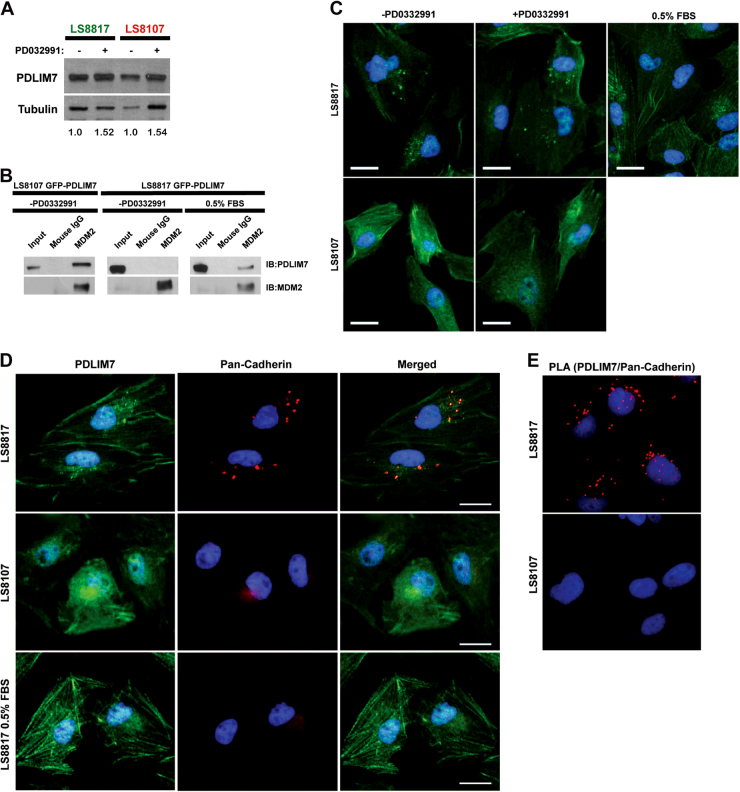
PDLIM7 is associated with MDM2 uniquely in LS8107 cells that quiesce and located in foci only in LS8817 cells that senesce. **a** The responder cell line LS8817 and non-responder cell line LS8107 were treated with 1 μM PD0332991 (PD) for 7 days. Cells were harvested for protein and MDM2 and PDLIM7 protein levels were detected using immunoblot. Tubulin served as a loading control. A representative image is shown. The mean expression value (PDLIM7/tubulin) quantified from four independent experiments is shown below each lane. **b** LS8817 and LS8107 cells were transduced with a vector expressing N-terminally tagged GFP-PDLIM7 and selected in puromycin. LS8817 cells were grown in serum-starved conditions with 0.5% serum for 4 days. MDM2 was immunoprecipitated and PDLIM7 immunoblotted (*n* = 3). IgG served as a control. **c** LS8817 and LS8107 cells were treated with either 1 μM PD0332991 (PD) for 7 days or grown in serum-starved conditions (0.5% serum) for seventy 2 h as shown. PDLIM7 was visualized by immunofluorescence (*n* = 3). **d** LS8817 and LS8107 cells were treated as shown. PDLIM7 and pan-cadherin were visualized by co-immunofluorescence. **e** LS8817 and LS8107 cells were fixed and incubated with antibodies against PDLIM7 and pan-cadherin followed by antibodies designed for the Sigma Duolink proximity ligation assay. Signal was visualized by immunofluorescence (*n* = 3)

PDLIM7 is a PDZ and LIM domain-containing protein that can bind MDM2 and inhibits its autoubiquitination, but does not block E3 ligase activity, allowing MDM2/PDLIM7 complexes to catalyze the ubiquitination of other substrates [22]. PDLIM7 was readily detectable in extracts from both non-responder LS8107 and responder LS8817 cells, and the amount was not affected by treatment with PD0332991 (Fig. 2a).

LS8817 cells are derived from a different patient with WD/DDLS. The addition of PD0332991 induces these cells to exit the cell cycle and undergo senescence, with concomitant decreases in the level of MDM2, increases in the number of ATRX foci, and accumulation of SA-β-gal and SAHF-positive cells. On the other hand, serum starvation will induce these cells to exit the cell cycle, but the expression of MDM2 is not decreased, the number of ATRX foci does not increase, and SA-β-gal and SAHF-positive cells do not accumulate [17, 18].

We thus determined whether the binding of PDLIM7 to MDM2 was associated with outcome. To accomplish this, we transduced LS8107 and LS8817 cells with a N-terminally green fluorescent protein (GFP)-tagged PDLIM7, treated the cells as indicated and immunoprecipitated MDM2. Tagging PDLIM7 was necessary to facilitate its identification by immunoblot because the immunoglobulin heavy chain obscures the endogenous protein. GFP-PDLIM7 coprecipitated with MDM2 in both cycling LS8107 cells and serum-starved LS8817 cells, but not in the cycling LS8817 cells (Fig. 2b). Similar results were observed in both cell lines transduced with a C-terminally GFP-tagged PDLIM7. This suggested that the interaction of PDLIM7 with MDM2 was both cell line and condition dependent.

### The cytosolic distribution of PDLIM7 is cell line and condition dependent

Although the expression of PDLIM7 could not account for the difference in MDM2 regulation following CDK4/6 inhibition in these two cell lines, the interaction of PDLIM7 with MDM2 was associated with the growth condition of LS8817 cells. PDZ domain proteins associate with actin and cytoskeletal structures [25–27], thus, we asked whether the localization of PDLIM7 might be associated with its ability to interact with MDM2. Immunofluorescence revealed that the localization of PDLIM7 was dynamically controlled. Focal depositions of cytosolic PDLIM7 were detected in the cycling LS8817 cells and in LS8817 cells that had undergone senescence following treatment with PD0332991, but not in quiescent serum-starved LS8817 cells (Fig. 2c). Focal depositions were also not detected in either cycling or PD0332991-treated quiescent LS8107 cells (Fig. 2c).

We next asked if other structural elements in the cell had a similar staining pattern in the two cell lines. There was no focal staining with antibodies raised against actin, γ-tubulin, or vimentin in either LS8817 or LS8107 cells (Supplemental Fig. 2). However, cytoplasmic foci were detected in LS8817 but not LS8107 cells stained with a pan-cadherin antibody (Supplemental Fig. 2).

Dual immunofluorescence indicated that the cytoplasmic cadherin foci overlapped with the PDLIM7 foci in LS8817 cells. No PDLIM7 or cadherin foci were identified in the LS8107 cells or serum-starved LS8817 cells (Fig. 2d). Confirming that these proteins were within 40 nm of each other we observed a positive signal in a proximity ligation assay (PLA) [28] in LS8817 cells (Fig. 2e). No such signal was observed when LS8817 cells were stained with individual antibodies or in LS8107 cells (Fig. 2e).

### PDLIM7 is associated with CDH18 in foci

The surprising appearance of cadherin reactivity in the cytoplasm of the cell prompted us to investigate this further. The peptide immunogen used to generate the pan-cadherin antibody is found throughout the cadherin-C superfamily (Supplemental Fig. 3A) [29, 30]. To identify the specific cadherin responsible for this staining pattern we knocked down several individual family members using at least two independent lentiviral shRNA-targeting vectors in LS8817 cells and screened for pan-cadherin foci by immunofluorescence. Only those shRNAs targeting CDH18 consistently reduced the number of foci to less than five per cell and increased the number of cells with no foci to ~ 20% (Supplemental Fig. 3B).

**Fig. 3:**
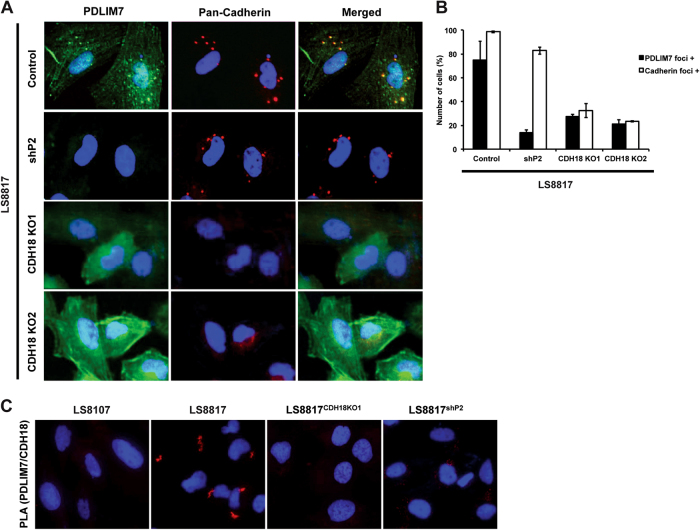
PDLIM7 is associated with CDH18 in foci in LS8817 cells. **a** The responder cell line LS8817 was unmanipulated (control), transduced with a PDLIM7 knockdown lentiviral vector (shP2), or a vector containing Cas9 and a vector containing a guide RNA against CDH18 (KO1 and KO2). PDLIM7 and pan-cadherin were visualized by co-immunofluorescence (*n* = 2). **b** The number of cells containing PDLIM7 and pan-cadherin foci were quantified. **c** Non-responder LS8107, responder LS8817, LS8817^KO1^, and LS8817^shP2^ cells were fixed and incubated with antibodies against PDLIM7 and CDH18 followed by antibodies designed for the Sigma Duolink proximity ligation assay. Signal was visualized by immunofluorescence (*n* = 2)

To validate the effect of CDH18 loss on the focal staining of PDLIM7 we generated two different CDH18 mutant clones of LS8817 cells using CRISPR/Cas9. These independent knockout cell lines, LS8817^CDH18KO1^ and LS8817^CDH18KO2^, targeted CDH18 exons one and five, respectively. Sanger sequencing confirmed the presence of indels that lead to predicted frameshifts (Supplemental Fig. 3C), and CDH18 protein was reduced in both clones compared with parental cells (Supplemental Fig. 3D). In both cell lines, pan-cadherin foci were reduced or lost (Figs. 3a, b). PDLIM7 foci were also lost in both clones (Figs. 3a, b). Further we were unable to detect positive PLA signals (Fig. 3c). Consequently, PDLIM7 interacted with CDH18 in these cytoplasmic foci.

### Knockout of CDH18 prevents PD0332991-induced MDM2 turnover and senescence

To determine whether differences in CDH18 expression might explain the differential response of cells to CDK4/6 inhibitors, we blotted CDH18 protein in a number of cell lines whose outcomes to CDK4/6 inhiibitors were previously characterized [18]. CDH18 protein was readily detected in LS8817, LS141, and LS0082 cells, and in the non-small cell lung cancer cell line H1975, all of which senesce following CDK4/6 inhibition (Supplemental Fig. 4). Expression of CDH18 was lower in LS8107 and LS8313 cells and in the non-small cell lung cancer cell line H358, all of which fail to senesce following CDK4/6 inhibition (Supplemental Fig. 4). All cells expressed PDLIM7, and although the amount varied, that did not correlate with the outcome of PD0332991 treatment, vis a vis quiescence or senescence (Supplemental Fig. 4).

**Fig. 4:**
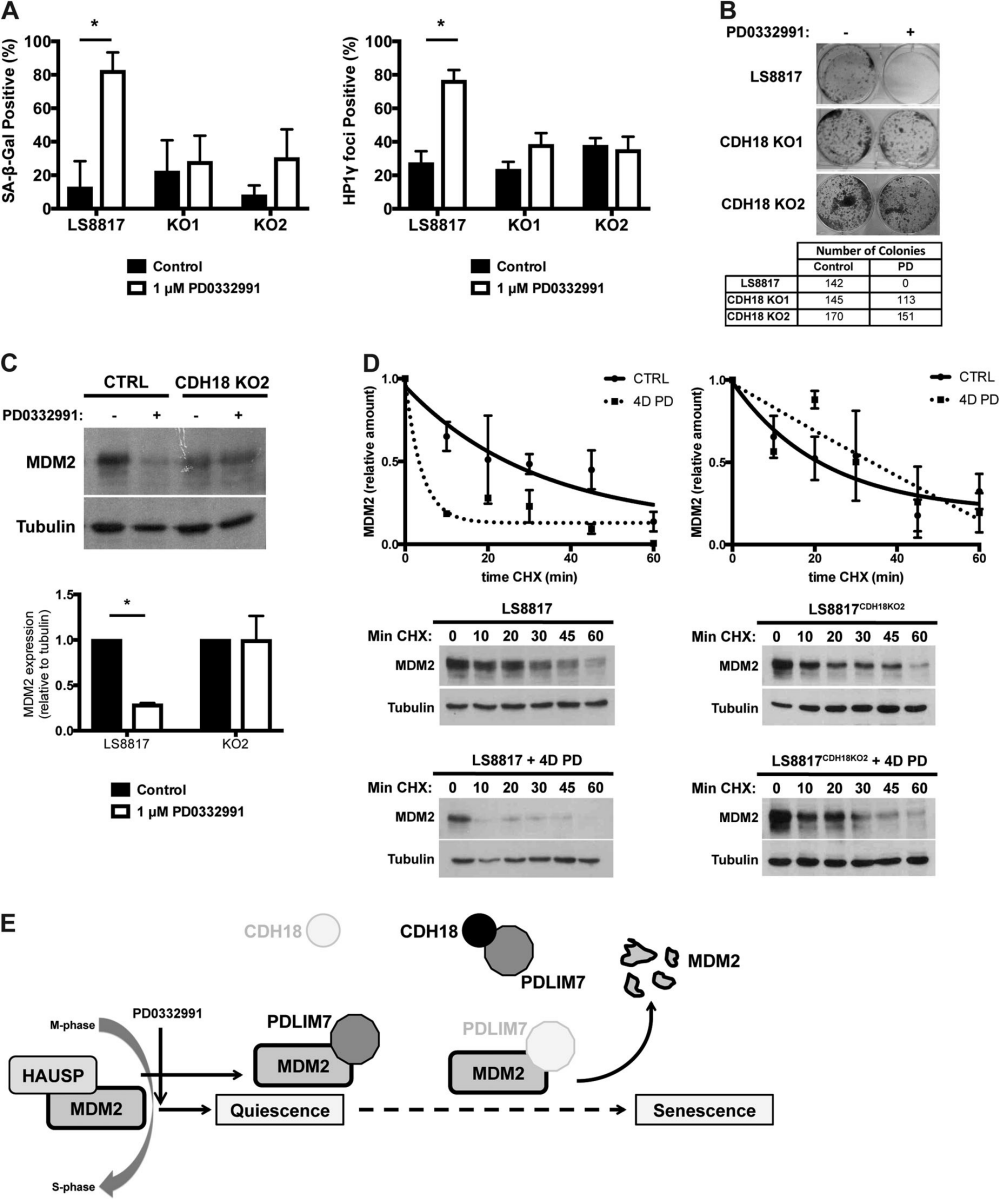
Knockout of CDH18 prevents CDK4 inhibitor-induced MDM2 turnover and senescence in LS8817 cells. **a** LS8817 cells and LS8817 cells transduced with the CDH18 knockout guide RNAs (KO1 and KO2) were treated with 1 μM PD0332991 (PD) for 7 days and the number of cells (mean ± standard deviation) staining for SA-β-gal and HP1γ foci were quantified (*n* = 3). **b** LS8817, LS8817 KO1, and LS8817 KO2 cells were treated with 1 μM PD0332991 for 10 days and then plated in drug-free media and allowed to grow for 21 days to assess clonogenic growth. A representative image from three biologic replicates is shown. **c** MDM2 protein levels were detected using immunoblot on extracts from LS8817 and LS8817 KO2 cells. Tubulin served as a loading control. Top, a representative image is shown. Bottom, expression was quantified using densitometry and the mean and standard deviation of three independent experiments is plotted. **d** LS8817 and LS8817 KO2 cells were treated with 1 μM PD for 4 days and treated with 75 μg/mL cyclohexamide (CHX) for the time (min) indicated. A representative image is shown (bottom) and relative amounts of MDM2 and tubulin quantified using densitometry and plotted (upper). **e** Summary of MDM2 regulation after CDK4 inhibition

To directly address whether CDH18 contributed to PD0332991-induced senescence and MDM2 turnover, we treated both LS8817 CDH18 knockout lines with PD0332991 and assayed a number of senescence markers seven days later. These lines were significantly impaired in their ability to undergo PD0332991-induced accumulation of SA-β-gal and SAHF (Fig. 4a). Furthermore, the LS8817^CDH18KO1^ and LS8817^CDH18KO2^ cells re-entered the cell cycle following removal of PD0332991 (Fig. 4b). MDM2 levels were not reduced in LS8817^CDH18KO2^ cells following treatment with PD0332991 (Fig. 4c), nor was the turnover of MDM2 accelerated to the same extent as seen in unmanipulated cells (Fig. 4d). Similar results were seen with LS8817^CDH18KO1^ cells. Collectively, this suggests that a CDH18 containing complex was sequestering PDLIM7 allowing for more rapid turnover of MDM2 and cellular senescence following treatment with PD0332991 (Fig. 4e).

### CDH18 expression in patients with WD/DDLS

In a small pilot study using seven pre- and post-treatment paired tumor sample biopsies from WD/DDLS patients who received palbociclib on clinical trial we had shown that the level of MDM2 was reduced in those who had a longer PFS [18]. This suggests that SAGA, induced by palbociclib, might be a mechanism that explains some of the clinical activity of this drug. To determine whether CDH18 might predict clinical benefit of the drug prior to therapy we measured its expression by immunohistochemistry in 49 surgical specimens obtained from patients who eventually enrolled in our palbociclib clinical trials and correlated this with PFS and OS.

**Fig. 5:**
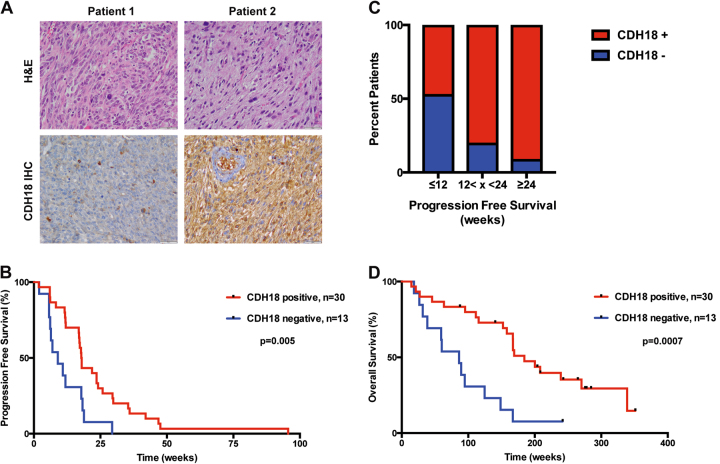
CDH18 expression can stratify patient response to palbociclib monotherapy. **a** Patient tumor samples were obtained during surgery and formalin fixed and paraffin embedded. After antigen retrieval, IHC was performed using a CDH18-specific antibody. Hematoxylin and eosin was used as a counterstain. Samples were blinded during analysis. Representative images are shown from two patients with dedifferentiated histologies, a CDH18-negative tumor (left) and a CDH18-positive tumor (right). **b** PFS was plotted for patients with CDH18-positive and CDH18-negative tumor samples (*p* = 0.005). **c** Patients were grouped based on their PFS ( ≤ 12 weeks, *n* = 18; 12 < × < 24 weeks, *n* = 14; ≥ 24 weeks, *n* = 11). The percent of patients in each group that were CDH18-positive are shown in red and the percent of patients that were CDH18-negative shown in blue. **d** OS was plotted for patients with CDH18-positive and CDH18-negative tumor samples (*p* = 0.0007)

The characteristics of these patients are shown in Supplemental Table 1. Details regarding the nature of the tissue samples and their staining with CDH18 antibodies are in the material and methods. The specificity of the antibody was validated in LS8817^CDH18KO^ cell lines. Blinded pathology review was carried out and reactivity described as positive or negative. Infiltrating inflammatory cells in negative samples served as a positive control for staining, whereas endothelial cells in blood vessels were a negative control in the positive samples (Fig. 5a). Five samples were censored as positive and negative staining tumor cells were localized into specific domains within the mass. Such intratumoral heterogeneity is consistent with the variation reported for genetic markers in liposarcoma [31], but makes interpretation difficult. One patient was censored because control positive infiltrating inflammatory cells were not detected in the section. Information on the histology of the disease at the time of surgery, dose and scheduling of palbociclib, prior therapies, duration of survival after starting palbociclib, and CDH18 reactivity for the remaining 43 individual patients analyzed is shown in Supplemental Fig. 5.

We plotted PFS (Figs. 5b, c) and OS (Fig. 5d) as a function of CDH18 expression in the remaining 43 patients. Patients whose tumors lacked CDH18 had worse outcomes than those who had CDH18 expression (PFS, *p* = 0.005; OS, *p* = 0.0007). Prior therapies and dosage schedule did not correlate with CDH18 staining and did not have a significant effect on either PFS or OS (Supplemental Table 2). However, all well-differentiated tumors were CDH18-positive, and because well-differentiated liposarcoma has a better prognosis than dedifferentiated liposarcoma [32], we reanalyzed the data after excluding well-differentiated samples. There was still a significant difference in outcome, with CDH18-positive patients having better survival, both by PFS (*p* = 0.05) and OS (*p* = 0.006) (Supplemental Figure 6).

For all CDH18-positive patients, the median PFS was 17.9 weeks (95% confidence interval, 17–25.9 weeks), and the median OS was 42.4 months (95% CI, 38.4 months to non-estimable (NE)). For CDH18-negative patients the median PFS was 9.0 weeks (95% CI, 6.1 weeks to NE) and OS was 19.8 months (95% CI, 8.9 months to NE). Although the power of the analysis is modest because the sample size is small, it does strengthen the hypothesis that SAGA is a clinically relevant biologic transition that underlies the efficacy of this class of drug.

## Discussion

MDM2 downregulation is necessary for CDK4/6 inhibitor therapy-induced senescence in a variety of cancer cell lines, and the loss of MDM2 is observed post palbociclib treatment in patients with WD/DDLS who have prolonged periods of PFS [18]. Given that senescence is a more desirable outcome of cytostatic therapy, we set out to define the mechanism controlling MDM2 turnover in response to PD0332991 in cell lines and then asked if this associates with the clinical outcome of WD/DDLS patients who were treated with palbociclib on a clinical trial. In this report, we show that the association of PDLIM7 with MDM2 can inhibit its turnover in cells that exit the cell cycle but fail to undergo senescence following exposure to PD0332991. In cells that do undergo senescence following drug treatment, PDLIM7 is sequestered in a complex with an intracellular cadherin, CDH18, in a serum-dependent manner. Furthermore, CDH18 expression correlates with the outcomes of patients with WD/DDLS who were treated with palbociclib, suggesting that SAGA is a bona fide mechanism of this drugs noted clinical activity.

There are two general outcomes of CDK4/6 inhibition-induced cell cycle exit in Rb-positive tumor cells. Cells can enter a quiescent state from which they are able to return to cycle following drug removal, or cells enter a senescent state from which they cannot return to cycle following drug removal [10–12]. Recently, it was shown that the decision of the cell to senesce follows upon its exit from the cell cycle, illuminating a new transition in cell cycle biology—senescence after growth arrest or SAGA.

The proteins required and the mechanisms by which they act during this transition in cells treated with CDK4/6 inhibitors are being actively pursued. MDM2 downregulation is necessary for this transition. Its E3 ligase activity is required to maintain the quiescent state. However, this is not because p53 is targeted by MDM2. Mutations affecting MDM2’s ability to bind to p53 do not affect its ability to suppress this transition, nor is p53 required for senescence induced by CDK4/6 inhibitors [13, 18].

Turnover of MDM2 during this transition is dependent on its own E3 ligase activity [18], and is prevented in cells that fail to senesce and remain in a quiescent state. HAUSP, a deubiquitinase, can bind to MDM2 [21]. HAUSP binding stabilizes MDM2 and allows it to ubiquitinate other substrates while removing the ubiquitins it places on itself. Nevertheless, HAUSP dissociates from MDM2 as the cells exit the cell cycle following treatment with PD0332991 regardless of the outcome [18], thus whatever prevents turnover in the quiescent cells must act downstream of this to stabilize MDM2. We show here that PDLIM7 binds to MDM2 inhibiting its turnover and preventing quiescent cells from progressing into senescence.

PDLIM7 had been previously reported to bind to MDM2, preventing its autoubiquitination and allowing MDM2 to trans-ubiquitinate p53 [19, 22]. Although PDLIM7 is ubiquitously expressed in the liposarcoma and non-small cell lung cancer cell lines we examined here, its association with MDM2 was cell type and condition specific. PDLIM7 could be sequestered in CDH18 complexes. Thus, sequestration of PDLIM7 in CDH18 complexes is a mechanism to control SAGA, after the dissociation of HAUSP from MDM2. Serum starvation can prevent this interaction in LS8817 cells allowing them to maintain themselves in quiescence rather than undergo senescence. Although the details of this regulatory event are not yet defined, it is tempting to speculate that this reflects context specific transformation events that sensitize cancer cells to CDK4/6 inhibitors. In normal untransformed cells, CDH18 and PDLIM7 interactions are not readily detected and such cells do not undergo CDK4/6 inhibitor-induced senescence.

In patients with WD/DDLS who received palbociclib, CDH18 is significantly associated with PFS and OS. As a predictive marker for PFS, it is quite clear that those patients who are negative for CDH18 are more likely to do poorly. When we model the successful outcome of PFS as **≥** 24 weeks, as defined by the primary endpoint of the clinical trials, the negative predictive value associated with the absence of CDH18 staining is 92.3% (95% CI, 64.0–99.8%). In contrast the positive predictive value of CDH18 staining is 33.3% (95% CI, 17.3–52.8%). Thus, IHC for CDH18 may identify those patients who would benefit from clinical strategies aimed at augmenting SAGA, potentially through inhibitor based combination strategies sequentially targeting CDK4/6 and the Ras signaling pathway, another regulator of SAGA [17].

Successful expansion of CDK4/6 therapies will require pre-treatment biomarkers that are predictive of response and are applicable in a large number of diverse tumor types [33]. WD/DDLS is characterized by amplification of 12q13–15 [32], which contains both the genes encoding MDM2 and CDK4. In WD/DDLS cell lines, the level of MDM2 is generally higher in asynchronously growing cells that will undergo senescence than in those that undergo quiescence following treatment with CDK4/6 inhibitors (Supplemental Fig. 4). Likewise, in the NSCLC cell lines we have looked at the level of MDM2 is generally higher in the growing cells that will undergo senescence than those that undergo quiescence (Supplemental Fig. 4). However, in neither cell type is it the level of MDM2 that determines the outcome—rather it is the reduction of that level from cycling to non-cycling that is associated with outcome. Indeed, a number of non-mesenchymal cell lines that do not have MDM2 amplification are induced to senesce by palbociclib treatment. Palbociclib can be used to successfully treat non-MDM2 amplified tumors, the most notable being HR+ Her2− breast cancers [34].

Remarkably, the twofold increase in the median PFS between CDH18-positive and CDH18-negative patients with WD/DDLS is comparable in magnitude to the twofold increase in the duration of response in HR + , Her2− breast cancer patients treated with letrozole and palbociclib. As of this writing, the only pre-treatment indication of response to one of the CDK4/6 inhibitor drugs, abemaciclib, is a signature of cyclin D activation, called DCAF [35, 36]. This is exclusively in cell lines. Our data suggest that CDH18 and a deeper understanding of SAGA might also predict if cell lines or a patient’s tumor would be innately resistant or clinically sensitive to CDK4/6 inhibitors.

## Materials and Methods

### Clinical Trials

The design and approvals of clinical trials with palbociclib have been previously described [23, 24]. Surgical resections were available from Memorial Hospital under IRB 10-094. PD0332991 was obtained from Selleckchem and used for the *in vitro* experiments.

### Cell lines

The WD/DDLS cell lines used in this manuscript were generated by Samuel Singer’s laboratory and previously described [18, 37]. Culture conditions and the response of these cell lines to CDK4 inhibition were previously described [17, 18].

### Lentivirus constructs

As described previously [18], lentivirus vectors were generated in 293 T cells by triple transfection with the vector of interest, psPAX2, and pMD2.G. Infected cells were selected using puromycin (1 μg/mL) or blasticidin (3 μg/mL) as appropriate. shRNA were delivered in the pLKO.1 vector (Open Biosystems). shRNA sequences are as follows: PDLIM7 (GCGAGACTATGAGAAGATGTT and CGTCTGTGCGATATGTCAGAT), CREBBP (CCCGATAACTTTGTGATGTTT and GCTATCAGAATAGGTATCATT), NEDD4-1 (GCCTTTCTCTTGCCTGCATAT and CGGTTGGAGAATGTAGCAATA), PKCD (CGGCATGAATGTGCACCATAA and CAGAGCCTGTTGGGATATATC), and P300 (CCAGCCTCAAACTACAATAAA and CCCGGTGAACTCTCCTATAAT). To rescue knockdown, cells were infected with a lentivirus encoding His-Biotin tagged PDLIM7 with a mismatched sequence.

### Real-time quantitative PCR

RNA was extracted from cells using the QIAGEN RNeasy kit per manufacturer’s instructions. Complementary DNA (cDNA) was synthesized from 1 μg of each RNA sample using the One *Taq* RT-PCR Kit and oligo-dT primers (New England BioLabs). cDNA was diluted 1:5 and 1 μL was used for qPCR per reaction. qPCR was performed by using 400 nM of each forward and reverse primer and SYBR Green PCR Master Mix (Life Technologies). qPCR was performed on Viia 7 Real-Time PCR System (Thermo Scientific). Primer sequences are as follows: PDLIM7 (Fw- CAGAGCCGCACCTCCATTG, Rev- TGGTGACACACGGGAGTCT), CREBBP (Fw- CCTGCCACGTCACAGACTG, Rev- GGCCAGAGTTACTATTGAGGAGG), p300 (Fw- GCTTCAGACAAGTCTTGGCAT, Rev- ACTACCAGATCGCAGCAATTC, PKCD (Fw- GTGCAGAAGAAGCCGACCAT, CCCGCATTAGCACAATCTGGA), NEDD4-1 (Fw- TCCAATGATCTAGGGCCTTTACC, Rev- TCCAACCGAGGATCTTCCCAT), β-actin (Fw- CATGTACGTTGCTATCCAGGC, Rev- CTCCTTAATGTACGCACGAT).

### Immunoblots

Cells were lysed with a buffer containing 50 mM Tris-HCl, pH 7.4, 250 mM NaCl, 5 mM thylenediaminetetraacetic acid, 0.5% NP40, 2 mM phenylmethylsulfonyl fluoride, and protease inhibitors. Depending on the antibody used forty to eighty micrograms of protein were resolved by sodium dodecyl sulfate polyacrylamide gel electrophoresis and transferred to polyvinylidene difluoride membranes. Membranes were incubated overnight with primary antibodies.

The antibodies used for immunoblotting were MDM2 (Santa Cruz Biotechnology SMP14) 1:500, CDK4 (Santa Cruz Biotechnology C-22) 1:1000, PDLIM7 (Santa Cruz Biotechnology H-110) 1:2000, CDH18 (Abnova 6F7) 1:500, Tubulin (Santa Cruz Biotechnology C-11) 1:2000.

Protein expression was quantified by densitometry analysis using ImageJ software.

### Immunoprecipitation

LS8817 and LS8107 cells were transduced with an LT3 lentivirus encoding GFP-tagged PDLIM7, and expression was induced by adding 10 μg/mL doxycycline to the media 2 days before cells were harvested. In LS8107 cells, expression of GFP-PDLIM7 was approximately equal to that of the endogenous protein. In LS8817 cells, expression was fourfold greater than the endogenous protein. Immunoprecipitation was performed by incubating 1.0–1.5 mg of protein lysate with 15–20 μL MDM2 SMP14 antibody or a mouse IgG control antibody overnight rotating at 4°C. Immune complexes were captured on either 30 μL of protein G sepharose or 20 μL of protein G dynabeads (catalog number 1003D) and subsequently eluted with 20–30 μL of 2 × sample buffer.

### Senescence assays

All senescence assays were done 7–10 days after drug treatment and quantified as described previously [17, 18].

### Immunofluorescence

Cells were plated in four-well glass chamber slides (Lab-Tek). Slides were fixed with 4% paraformaldehyde for 15 min, permeabilized with 0.1% triton for 5 min, blocked with phosphate-buffered saline (PBS) /0.5% Tween-20/1% bovine serum albumin for 20 min, and primary antibody was diluted in blocking buffer overnight. Dilutions for antibodies were as follows: HP1γ (Cell Signaling 05–690) 1:5000, ATRX (Santa Cruz Biotechnology H-300) 1:2000, pan-Cadherin (BD Biosciences 610181) 1:1000, PDLIM7 (Santa Cruz Biotechnology H-110) 1:2000. Fluorescent secondary antibodies (Alexa Flour Rabbit 488 and Mouse 546) were diluted at 1:500 and incubated for 1 h at room temperature and slides were counterstained with Hoechst for nuclear visualization. The Duolink Proximity Ligation Assay (Sigma) was performed according to manufacturer’s instructions.

### CRISPR Cas9

lentiCas9-Blast and lentiGuide-Puro were a ‘gift’ from Feng Zhang (Addgene plasmid numbers 52962 and 52963). Guide sequences targeting CDH18 were designed using Benchling software. Guides were tested for successful genomic editing by the T7 endonuclease mismatch assay as described previously [38]. Guides were transduced into LS8817 cells and selected in puromycin for 6 weeks. Clones that demonstrated efficient cutting were sequenced and tested for the loss of CDH18 protein as measured by immunoblot. Cells were maintained in culture for up to eight weeks after sequencing while experiments were carried out.

### Immunohistochemistry

Formalin-fixed paraffin embedded blocks were sectioned at four microns. The well-differentiated or dedifferentiated status of the tumor and tumor cells were identified by visualization of a parallel section stained by Haemotoxylin and Eosin. Tissue sections ranged from 200 mm^2^ to 600 mm^2^ (with an average of 377 mm^2^) and were obtained from surgical resections carried out 100–2100 days prior to palbociclib therapy. Immunohistochemistry detection with CDH18 antibody was performed using the Discovery XT processor (Ventana Medical Systems). Tissue sections were blocked first for 30 min in MOM Block (Vector Labs, MKB-2213) in PBS. A mouse monoclonal antibody to CDH18 (Abnova, H00001016-M01) was used at a 3 μg/mL concentration and incubation was done for 4 h, followed by another 60 min incubation with biotinylated anti-mouse Secondary (Vector Labs, MOM Kit BMK-2202), at a 5.75 μg/mL concentration. Blocker D, Streptavidin-horseradish peroxidase and DAB detection kit (Ventana Medical Systems) were used according to the manufacturer instructions. Tumor cells were identified within the sample based on their nuclear morphology. Samples where > 80% of the tumor cells throughout contained cytoplasmic CDH18 staining were deemed positive.

Twenty-eight of the 49 patients received at least one prior systemic therapy sometime between this surgery and treatment with palbociclib. These included gemcitabine, doxil, docetaxel, brivanib, irinotecan, or an MDM2 inhibitor, either alone or in various combinations. The median number of therapies received was one (range from one to four). Two different dose schedules of palbociclib were used. Thirty-two patients received palbociclib at 125 mg daily for 21 days followed by a rest period of 7 days every 28 days, and 17 received 200 mg daily for 14 days with a rest period of 7 days every 21 days.

### Statistical analysis

Disease progression and death were the endpoints of this study. Disease progression was defined as the time from the treatment start to the occurrence of disease progression; death was defined as the time from the treatment start to the occurrence of death owing to any cause or the date of last follow-up. The clinicopathologic variables examined were CDH18 staining (positive vs. negative), treatment dosage level (125 mg vs. 200 mg), disease subtype (well-differentiated vs. dedifferentiated), and prior therapy (yes vs. no). The probabilities of progression and death were each estimated using the Kaplan–Meier method. The associations of these outcomes with the clinicopathologic variables were examined using the log-rank test for categorical variables. *P* values ≤ 0.05 were considered significant. All analyses were performed using R version 3.2.0 (cran.r-project.org).

## Electronic supplementary material

List of supplementary material

Supplemental figure legends

Supplemental Table 1

Supplemental Table 2

Supplemental Figure 1

Supplmental Figure 2

Supplemental Figure 3

Supplemental Figure 4

Supplemental Figure 5

Supplmental Figure 6
