# Supplementary material for: PDLIM7 and CDH18 regulate the turnover of MDM2 during CDK4/6 inhibitor therapy-induced senescence
Source: Oncogene. 2018 May 23;37(37):5066–78. doi: 10.1038/s41388-018-0332-y (PMC6137027; doi:10.1038/s41388-018-0332-y)
Supplement: Supplementary file 1 — List of supplementary material [file 41388_2018_332_MOESM1_ESM.docx]

**LIST OF SUPPLEMENTAL MATERIALS**

**Supplemental Table 1**. Overall Patient Characteristics

**Supplemental Table 2**. Patient Characteristics based on CDH18 staining status

**Supplemental Figure 1.** PDLIM7 knockdown allows PD0332991 to induce accumulation of SA-β-gal in non-responder LS8313 cells

**Supplemental Figure 2**. Immunofluorescence staining of cytoskeletal elements in LS8817 responder and LS8107 non-responder cells

**Supplemental Figure 3.** Knocking down CDH18 can reduce the appearance of pan-cadherin foci in LS8817 cells

**Supplemental Figure 4.** CDH18, PDLIM7, MDM2 and CDK4 immunoblots in the cell lines used in this study

**Supplemental Figure 5.** CDH18 expression and characteristics of the patient samples used in this study

**Supplemental Figure 6.** CDH18 expression stratifies response to monotherapy palbociclib in dedifferentiated tumors alone.
