# Supplementary material for: PDLIM7 and CDH18 regulate the turnover of MDM2 during CDK4/6 inhibitor therapy-induced senescence
Source: Oncogene. 2018 May 23;37(37):5066–78. doi: 10.1038/s41388-018-0332-y (PMC6137027; doi:10.1038/s41388-018-0332-y)
Supplement: Supplementary file 2 — Supplemental figure legends [file 41388_2018_332_MOESM2_ESM.docx]

**Supplemental Figure 1. PDLIM7 knockdown allows PD0332991 to induce accumulation of SA-β-gal in non-responder LS8313 cells.**  The non-responder cell line LS8313 was transduced with a PDLIM7 knockdown lentiviral vector (shP2) or a non-specific vector (scr). Cells were then transduced with a his-biotin tagged PDLIM7 wobble expression vector wherein the sequence was mutated to prevent recognition by the shRNA without effecting coding sequence. Cells were treated with 1 μM PD0332991 (PD) for 7 days and the number of cells staining for SA-β-gal were quantified. The mean and standard error of measurement from two experiments is plotted. PDLIM7 protein levels were measured using immunoblot. Tubulin served as a loading control. The mean relative amount (PDLIM7/tubulin) from two independent experiments is quantified blow each lane.

**Supplemental Figure 2. Immunofluorescence staining of cytoskeletal elements in LS8817 responder and LS8107 non-responder cells.** LS8107 and LS8817 cells were fixed, permeabilized, and independently incubated with antibodies against PDLIM7, actin, γ-tubulin, vimentin and pan-cadherin. Slides were incubated with fluorescent secondary antibodies and visualized using immunofluorescence. Representative images from three independent experiments are shown.

**Supplemental Figure 3. Knocking down CDH18 prevents foci can reduce the appearance of pan-cadherin foci in LS8817 cells.** (A) Schematic of a type I cadherin (CDH1) and a type II cadherin (CDH18). Homology to the pan-Cadherin peptide immunogen for BD biosciences 610181 is shown. (B) LS8817 cells were transduced with independent short hairpins against the cadherin targets indicated and selected in puromycin for 5 days. The cells were fixed, permeabilized, and incubated with antibodies against pan-cadherin. The number of cells containing greater than 5 cadherin foci and 0 cadherin foci were quantified in at least five 20X fields for each knockdown (100-500 cells total). (C) LS8817 cells were transduced with a vector containing Cas9 and a vector containing a guide RNA against CDH18 (KO1 and KO2). Targeting sequencings are shown. Cutting was confirmed by sequencing. (D) LS8817 cells transduced with the guide RNAs were harvested for protein. CDH18 protein levels were measured using immunoblot, Tubulin served as a loading control.

**Supplemental Figure 4. CDH18, PDLIM7, MDM2 and CDK4 immunoblots in the cell lines used in this study.** Proteins were extracted from asynchronously growing responder well differentiated/dedifferentiated liposarcoma cell lines LS8817, LS141, and LS0082 and non-small cell lung cancer cell line H1975 and the non responder well differentiated/dedifferentiated liposarcoma cell lines LS8107 and LS8313 and non-small cell lung cancer cell line H358 and expression levels of the indicated proteins determined by immunoblot. Tubulin served as a loading control. To quantify the blots, band intensities were measured in at least three independent experiments, and the relative amount of protein normalized by the amount of tubulin. The value for LS8817 and H1975 cells was set to 1.0 to allow comparison with other liposarcoma or NSCLC cell lines. The mean value of protein expression is then shown below each lane

**Supplemental Figure 5. CDH18 expression and characteristics of the patient samples used in this study.** Red (dedifferentiated histology) and purple (well-differentiated histology) hued bars represent the time (days) from the most proximal surgical resection to the time the patient began palbociclib (Rx). Asterisks denote when patients received therapies between the time of surgical resection and before palbociclib treatment began. Orange hued bars represent the time (days) that the patient was on palbociclib before they progressed as defined in our previous papers [1, 2]. The dosage of palbociclib received is represented by the hue of the bar (light orange, 125 mg protocol; dark orange 200 mg protocol). CDH18 reactivity by IHC is indicated to the right. Overall survival (OS) as of December 7, 2017 is indicated to the right.

**Supplemental Figure 6. CDH18 expression stratifies response to monotherapy palbociclib in dedifferentiated tumors alone.** This is arranged as described in the legend to figure 5 but limited to only patients whose tumors were dedifferentiated at the time of surgical resection. (A) PFS. p=0.05. (B) OS. p=0.006.

1. Dickson MA, Schwartz GK, Keohan ML, D'Angelo SP, Gounder MM, Chi P, Antonescu CR, Landa J, Qin LX, Crago AM, Singer S, Koff A and Tap WD. Progression-Free Survival Among Patients With Well-Differentiated or Dedifferentiated Liposarcoma Treated With CDK4 Inhibitor Palbociclib: A Phase 2 Clinical Trial. JAMA oncology. 2016; 2(7):937-940.

2. Dickson MA, Tap WD, Keohan ML, D'Angelo SP, Gounder MM, Antonescu CR, Landa J, Qin LX, Rathbone DD, Condy MM, Ustoyev Y, Crago AM, Singer S and Schwartz GK. Phase II trial of the CDK4 inhibitor PD0332991 in patients with advanced CDK4-amplified well-differentiated or dedifferentiated liposarcoma. Journal of clinical oncology : official journal of the American Society of Clinical Oncology. 2013; 31(16):2024-2028.
