# Supplementary figures and images for: PDLIM7 and CDH18 regulate the turnover of MDM2 during CDK4/6 inhibitor therapy-induced senescence
Source: Oncogene. 2018 May 23;37(37):5066–78. doi: 10.1038/s41388-018-0332-y (PMC6137027; doi:10.1038/s41388-018-0332-y)

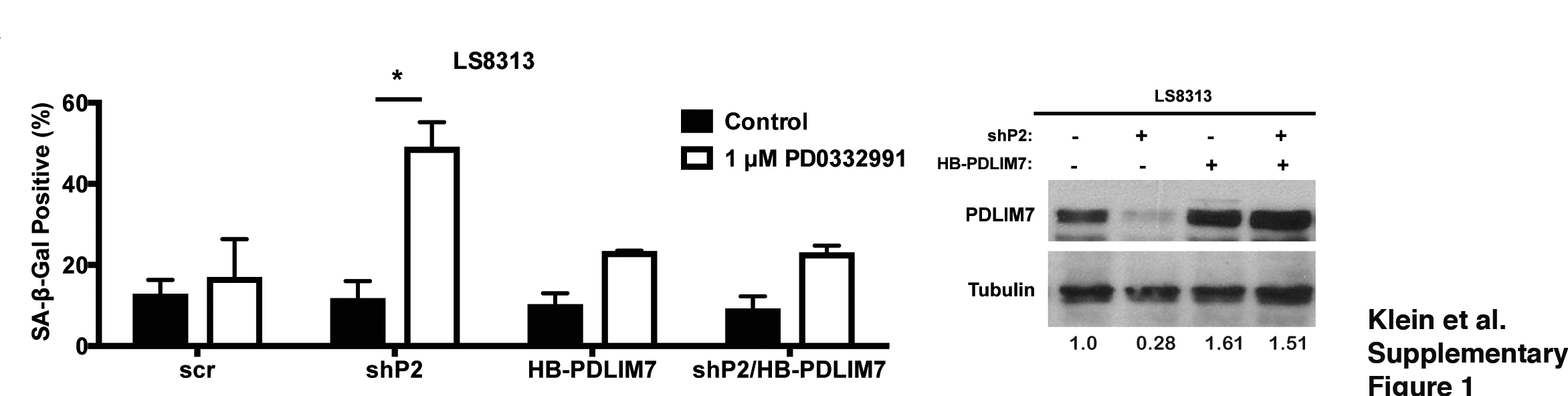

Supplement: Supplementary file 5 — Supplemental Figure 1 [file 41388_2018_332_MOESM5_ESM.tif]

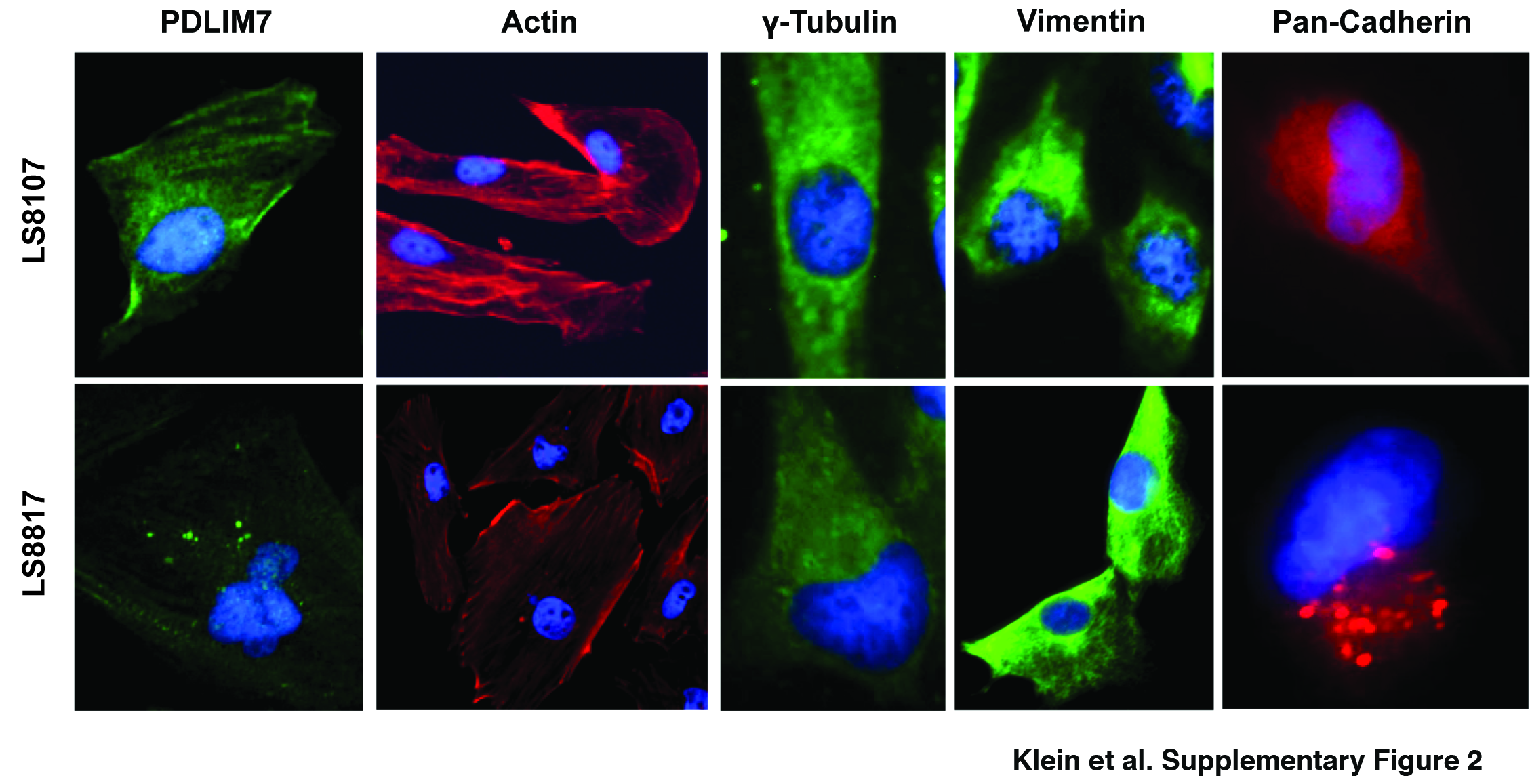

Supplement: Supplementary file 6 — Supplmental Figure 2 [file 41388_2018_332_MOESM6_ESM.tif]

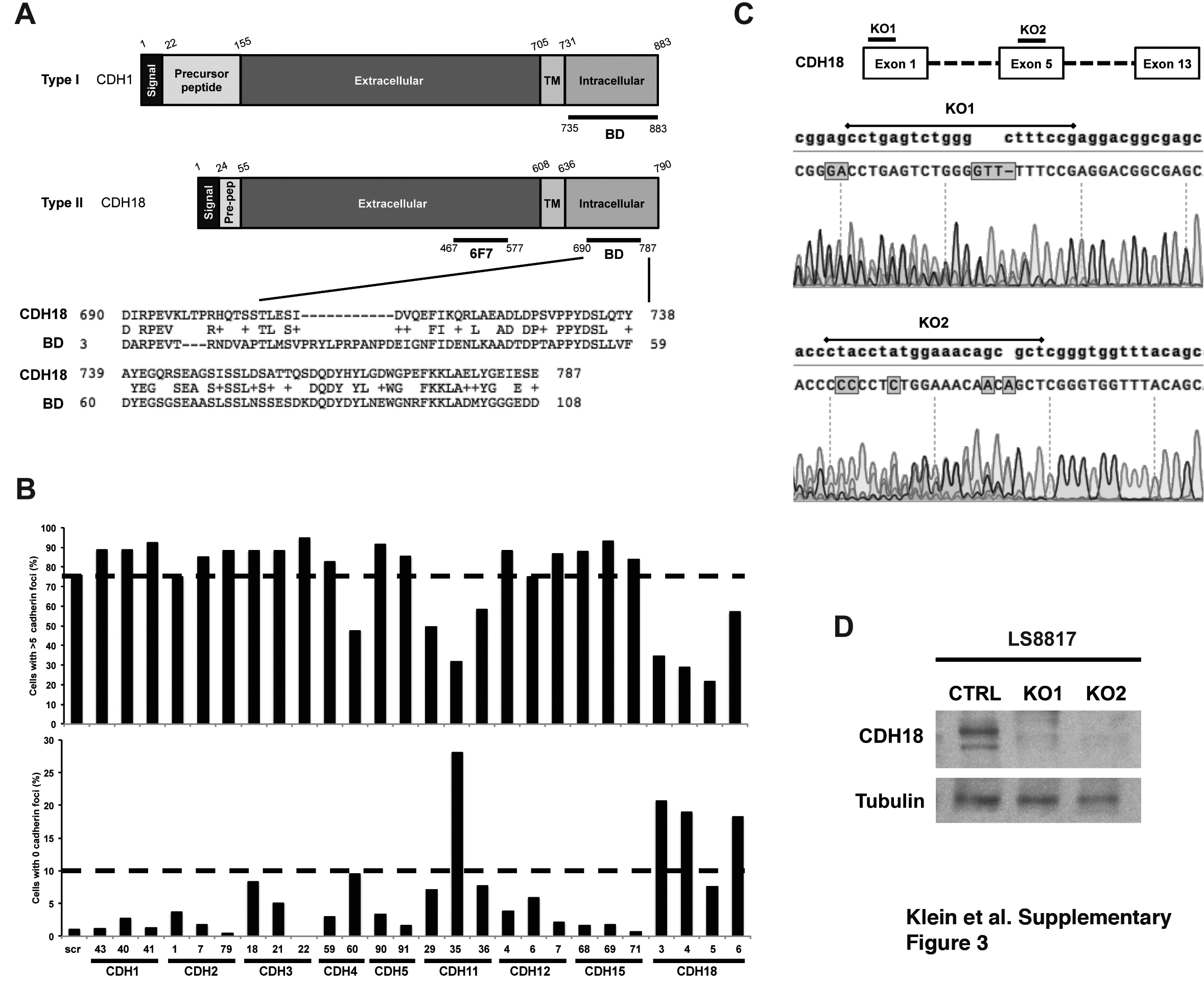

Supplement: Supplementary file 7 — Supplemental Figure 3 [file 41388_2018_332_MOESM7_ESM.tif]

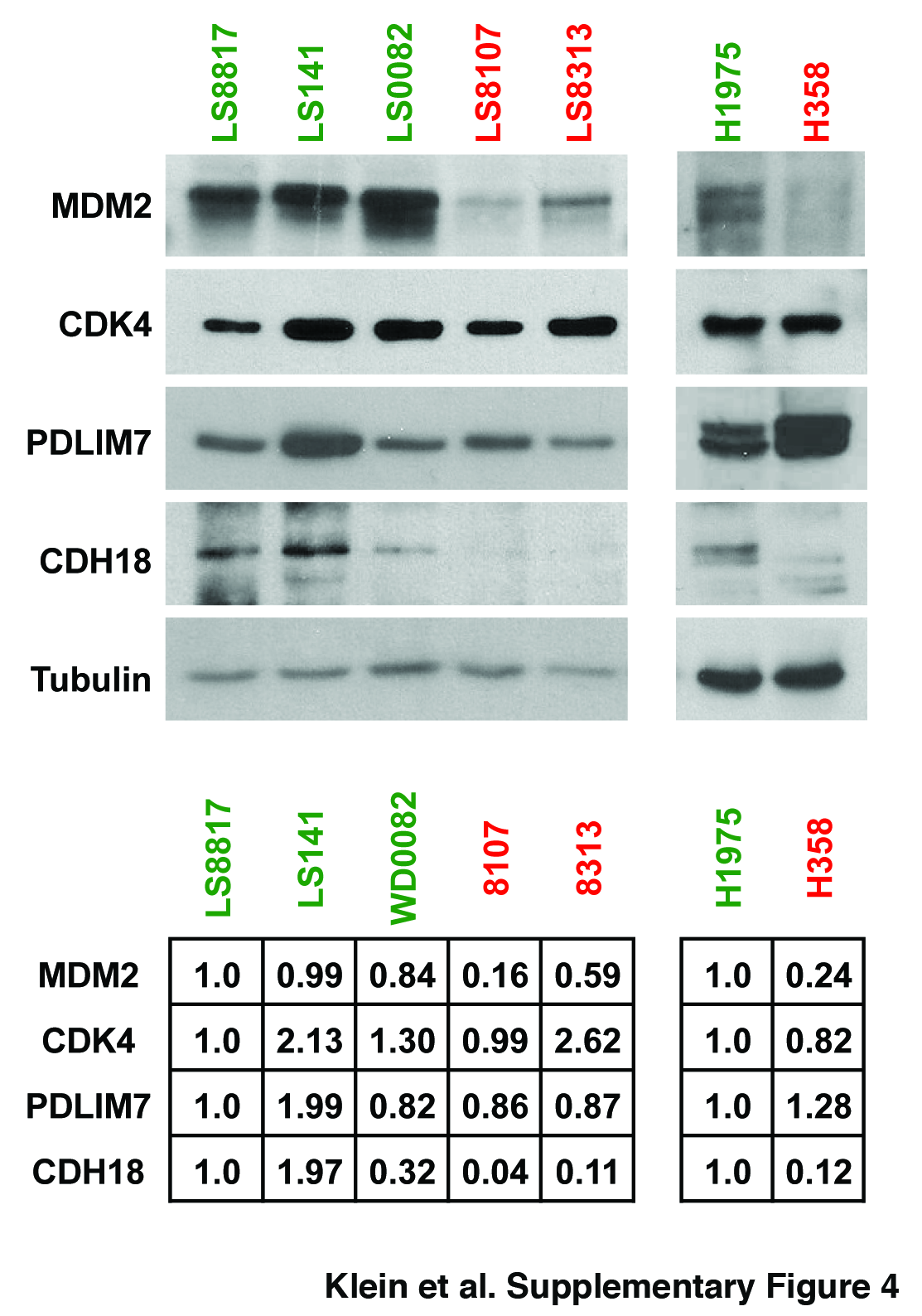

Supplement: Supplementary file 8 — Supplemental Figure 4 [file 41388_2018_332_MOESM8_ESM.tif]

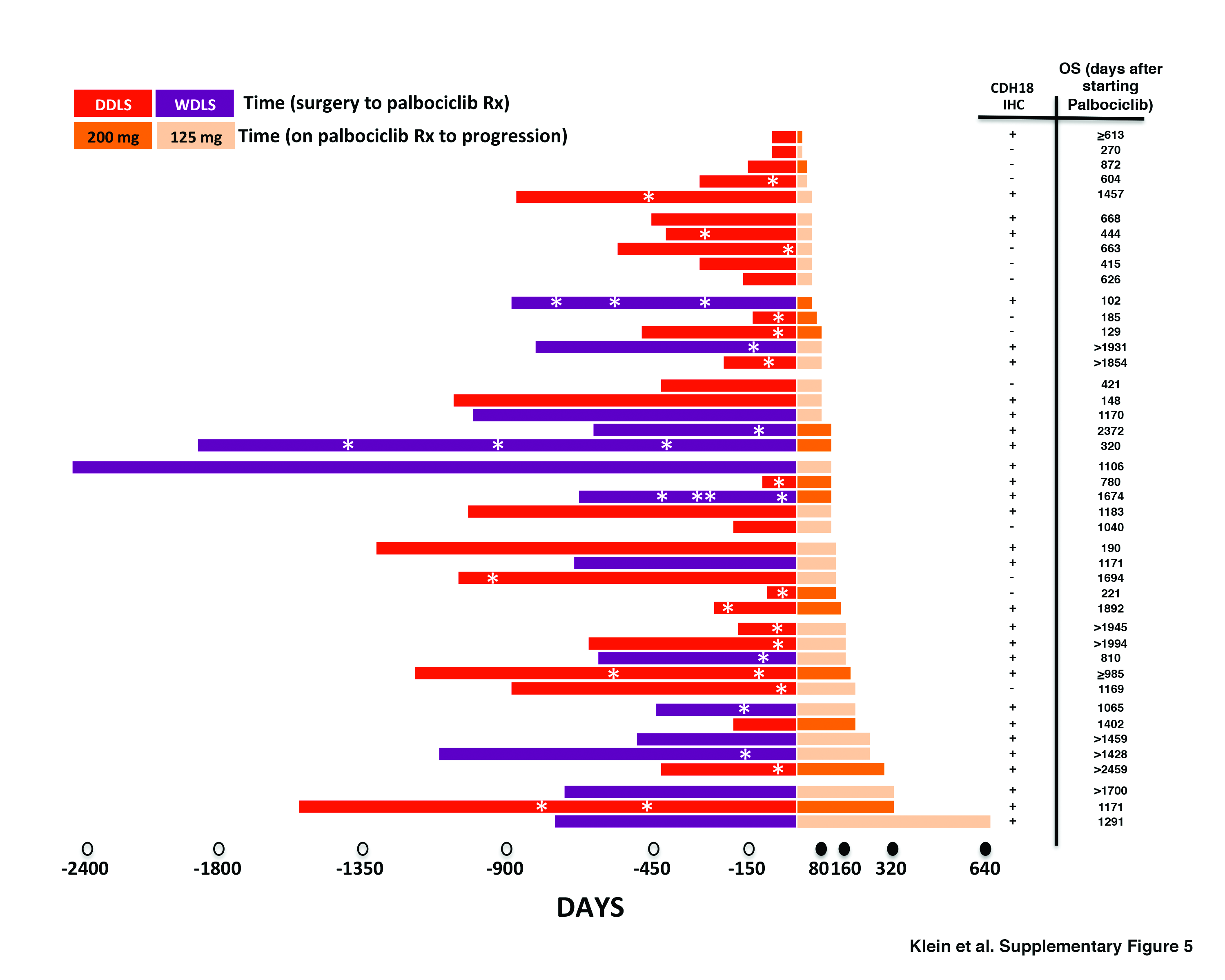

Supplement: Supplementary file 9 — Supplemental Figure 5 [file 41388_2018_332_MOESM9_ESM.tif]

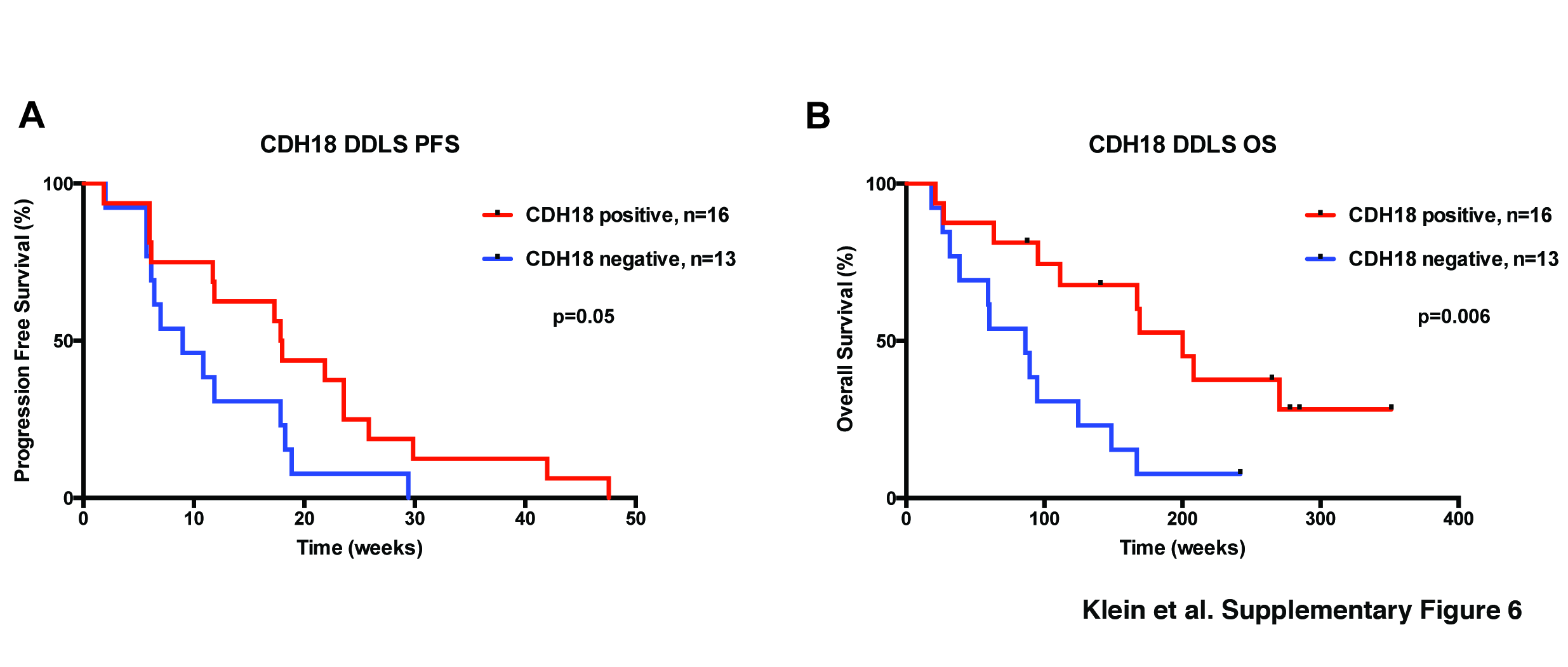

Supplement: Supplementary file 10 — Supplmental Figure 6 [file 41388_2018_332_MOESM10_ESM.tif]
