# Supplementary material for: PDLIM7 and CDH18 regulate the turnover of MDM2 during CDK4/6 inhibitor therapy-induced senescence
Source: Oncogene. 2018 May 23;37(37):5066–78. doi: 10.1038/s41388-018-0332-y (PMC6137027; doi:10.1038/s41388-018-0332-y)
Supplement: Supplementary file 4 [file 41388_2018_332_MOESM3_ESM.docx]

Supplementary Table 1. Overall Patient Characteristics

Characteristics		125mg cohort	200mg cohort	Total
Number		33	16	49
Male		15	10	25
Female		18	6	24
Median Age (range), y		64 (35-85)	70 (41-83)	65 (35-85)
ECOG Score				
	0	31	7	38
	1	2	9	11
Primary Site				
	retroperitoneum	32	16	48
	extremity	1	0	1
Histology				
	Well-differentiated	11	4	15
	Dedifferentiated	22	12	34
Prior Systemic Treatment, No				
	0	17	3	20
	1	16	7	23
	2+	0	6	6
