# Supplementary material for: PDLIM7 and CDH18 regulate the turnover of MDM2 during CDK4/6 inhibitor therapy-induced senescence
Source: Oncogene. 2018 May 23;37(37):5066–78. doi: 10.1038/s41388-018-0332-y (PMC6137027; doi:10.1038/s41388-018-0332-y)
Supplement: Supplementary file 5 [file 41388_2018_332_MOESM4_ESM.docx]

Supplementary Table 2. Patient Characteristics based on CDH18 status

Characteristics	CDH18-	CDH18+	Total
Number	13	30	43
Male	6	15	21
Female	7	15	22
Median Age (range), y	64 (41-83)	63 (35-81)	64 (35-83)
ECOG Score			
0	11	22	33
1	2	8	10
Primary Site			
retroperitoneum	12	30	42
extremity	1	0	1
Histology			
Well-differentiated	0	14	14
Dedifferentiated	13	16	29
Prior Systemic Treatment, No			
0	6	12	18
1	7	13	20
2+	0	5	5
Dosage schedule of palbociclib			
125 mg	9	19	28
200 mg	4	11	15
